# Impact of stress-induced hyperglycemia on the outcome of children with trauma: A cross-sectional analysis based on propensity score-matched population

**DOI:** 10.1038/s41598-019-52928-6

**Published:** 2019-11-08

**Authors:** Yi-Wen Tsai, Shao-Chun Wu, Chun-Ying Huang, Shiun-Yuan Hsu, Hang-Tsung Liu, Ching-Hua Hsieh

**Affiliations:** 1 Department of Pediatric Surgery, Kaohsiung Chang Gung Memorial Hospital, Chang Gung University and College of Medicine, Kaohsiung, 83301 Taiwan; 2 Department of Anesthesiology, Kaohsiung Chang Gung Memorial Hospital, Chang Gung University and College of Medicine, Kaohsiung, 83301 Taiwan; 3 Department of Trauma Surgery, Kaohsiung Chang Gung Memorial Hospital, Chang Gung University and College of Medicine, Kaohsiung, 83301 Taiwan; 4 Department of Plastic Surgery, Kaohsiung Chang Gung Memorial Hospital, Chang Gung University and College of Medicine, Kaohsiung, 83301 Taiwan

**Keywords:** Risk factors, Population screening

## Abstract

This was a retrospective study of pediatric trauma patients and were hospitalized in a level-1 trauma center from January 1, 2009 to December 31, 2016. Stress-induced hyperglycemia (SIH) was defined as a hyperglycemia level ≥200 mg/dL upon arrival at the emergency department without any history of diabetes or a hemoglobin A1c level ≥6.5% upon arrival or during the first month of admission. The results demonstrated that the patients with SIH (n = 36) had a significantly longer length of stay (LOS) in hospital (16.4 vs. 7.8 days, p = 0.002), higher rates of intensive care unit (ICU) admission (55.6% vs. 20.9%, p < 0.001), and higher in-hospital mortality rates (5.6% vs. 0.6%, p = 0.028) compared with those with non-diabetic normoglycemia (NDN). However, in the 24-pair well-balanced propensity score-matched patient populations, in which significant difference in sex, age, and injury severity score were eliminated, patient outcomes in terms of LOS in hospital, rate of ICU admission, and in-hospital mortality rate were not significantly different between the patients with SIH and NDN. The different baseline characteristics of the patients, particularly injury severity, may be associated with poorer outcomes in pediatric trauma patients with SIH compared with those with NDN. This study also indicated that, upon major trauma, the response of pediatric patients with SIH is different from that of adult patients.

## Introduction

Hyperglycemia is defined as serum glucose level >200 mg/dL. A positive correlation was observed between hyperglycemia upon admission and poor outcome in patients with trauma^1–4^. Hyperglycemia is not always attributed to diabetes mellitus, and in some cases, it may be secondary to stress^5^. Hyperglycemia may be part of the neuroendocrine response to stress in critically ill patients or those with trauma^6,7^. The hemodynamic response was described in the literature as a combination of high plasma catecholamine levels, increased sympathetic activity, and increased cortisol levels that regulate cardiac output, blood pressure, and end-organ perfusion^6,8,9^. These mechanisms may alter carbohydrate metabolism, which include increased gluconeogenesis, depressed glycogenesis, glucose intolerance, and insulin resistance, as a result of decreased glucose uptake in the skeletal muscle^10,11^.

Stress-induced hyperglycemia (SIH) is a type of hyperglycemia that is secondary to stress, and it commonly occurs in patients with trauma^1,2,10,12,13^ or with critical illness^14^. SIH, but not diabetic hyperglycemia, was associated with a significantly higher mortality risk in patients with trauma^13,15–17^. Moreover, SIH is associated with a 3-fold higher odds of mortality than non-diabetic normoglycemia (NDN) in patients with trauma^15^. However, studies about the outcome of patients with trauma who presented with SIH in pediatric patients were limited. Therefore, in this study, we aimed to assess the impact of SIH compared with NDN on the outcomes of pediatric patients with trauma in the selected propensity-score-matched patient population. This study primarily hypothesized that patients with SIH had a worse outcome than those with NDN.

## Methods

### Ethical statement

The institutional review board (IRB) of Chang Gung Memorial Hospital had preapproved this study before its initiation (approval number: 201800884B0). The need for informed consent was waived according to IRB regulations.

### Data source and study population

This retrospective study reviewed the data of all hospitalized patients with trauma who were registered in the Trauma Registry System of the hospital^18,19^ from January 1, 2009 to December 31, 2016. The patients were diagnosed with hyperglycemia if they presented with serum glucose level ≥200 mg/dL upon arrival to the emergency department (ED) based on the current criteria of the American Diabetes Association^20^, and SIH was defined as the presence of hyperglycemia without any history of diabetes or hemoglobin A1c (HbA1c) level ≥6.5% upon arrival or during the first month of admission^15–17^. NDN was defined as serum glucose levels <200 mg/dL upon arrival at the ED without history of diabetes or hemoglobin A1c (HbA1c) level ≥6.5% upon arrival or during the first month of admission. Only pediatric patients aged 2–19 years with available data about serum glucose levels upon admission were included in the study, and those patients with incomplete registered data were excluded from this study. A detailed patient information was retrieved, which included the following variables: sex, age, trauma mechanisms (driver of motor vehicle, passenger of motor vehicle, driver of motorcycle, passenger of motorcycle, bicycle, pedestrian, fall, and strike by/against object like assault or hit with wall or heavy object), associated injuries, abbreviated injury scale (AIS) score in different body regions, injury severity score (ISS), serum glucose level upon arrival at the ED, and complications such as pneumonia, urinary tract infection, wound infection, acute respiratory failure, upper gastrointestinal bleeding, and fat embolism. Isolated AIS ≥3 body region was also recorded, considering that a patient with AIS ≥3 in two or more different body regions was considered to have polytrauma^21^. The primary outcome of this study was in-hospital mortality, and the secondary outcome was length of stay (LOS) in the hospital and admission rate in the intensive care unit (ICU).

### Statistical analysis

The unpaired student’s *t*-test and the Mann–Whitney U test were used to analyze normally and non-normally distributed continuous data, which were expressed as mean with standard deviation and median with interquartile range (IQR: Q1–Q3), respectively. Categorical data were expressed as number and frequency (%) and were compared using two-sided Fisher’s exact or Pearson’s chi-square tests with the presentation of odds ratios (ORs) with 95% confidence intervals (CIs). To minimize the confounding effects of sex, age, and injury severity of patients on outcome measurements, a logistic regression model was used to calculate the propensity scores with the following covariates: sex, age, and ISS. Subsequently, 1:1-propensity score-matched patient populations were established using the Greedy method with a 0.2-caliper width using the NCSS 10 software (NCSS statistical software, Kaysville, UT, the USA) for the assessment of the impact of SIH compared with NDN on the outcomes. All statistical analyses were performed using the Statistical Package for the Social Sciences software for Windows version 22 (IBM Corp., Armonk, NY, the USA). *p*-values less than 0.05 were considered statistically significant.

## Results

### Characteristics of patients with SIH and NDN

A total of 1,058 non-diabetic patients with trauma, aged 2–19 years, were included in the study. The patients were divided into two groups: SIH group (n = 36) and NDN group (n = 1,022). As shown in Table 1, no significant difference was observed in terms of sex and age between patients with SIH and NDN. Head trauma is most frequently associated with SIH, followed by injuries to the extremity, abdomen, and thoracic regions (Table 2). Regarding the injured body regions with an AIS score ≥3, significantly higher rates were observed for head/neck, thorax, abdomen, and external injuries in patients with SIH compared with those with NDN (Table 1). Regarding the body regions with an isolated AIS score ≥3, significantly higher rates were only observed for external injuries, but not for head/neck, thorax, and abdomen injuries, in patients with SIH than those with NDN. In addition, patients with SIH had significantly higher ISS than those with NDN (median [IQR]: 17.5 [9.0–28.3] vs. 5.0 [4.0–10.0]; p < 0.001). When stratified by injury severity (ISS < 16, 16–24, or ≥25), the number of patients with SIH who had an ISS of 16–24 and ≥25 was higher, and the number of patients with SIH who had an ISS <16 was lower than that of patients with NDN.

**Table 1:** Characteristics of injury and pediatric trauma patients who presented with stress-induced hyperglycemia (SIH) and non-diabetic normoglycemia (NDN).

Variables	SIH (n = 36)	NDN (n = 1,022)	P
Sex, n (%)			0.855
Male	24(66.7)	707(69.2)	
Female	12(33.3)	315(30.8)	
Age, years	12.6 ± 5.7	14.4 ± 4.9	0.073
Mechanisms, n (%)			
Driver of motor vehicle	0(0.0)	5(0.5)	1.000
Passenger of motor vehicle	0(0.0)	12(1.2)	1.000
Driver of motorcycle	12(33.3)	442(43.2)	0.304
Passenger of motorcycle	6(16.7)	99(9.7)	0.249
Bicycle	3(8.3)	86(8.4)	1.000
Pedestrian	4(11.1)	17(1.7)	0.004
Fall	8(22.2)	232(22.7)	1.000
Strike by/against object	3(8.3)	129(12.6)	0.610
AIS ≥3, n (%)			
Head/Neck	17(47.2)	192(18.8)	<0.001
Face	1(2.8)	3(0.3)	0.129
Thorax	5(13.9)	35(3.4)	0.009
Abdomen	9(25.0)	39(3.8)	<0.001
Extremity	11(30.6)	228(22.3)	0.309
External	2(5.6)	4(0.4)	<0.001
Isolated AIS ≥3, n (%)			
Head/Neck	9(25.0)	164(16.0)	0.153
Face	0(0.0)	2(0.2)	0.790
Thorax	1(2.8)	12(1.2)	0.391
Abdomen	2(5.6)	22(2.2)	0.178
Extremity	4(11.1)	205(20.1)	0.185
External	2(5.6)	4(0.4)	<0.001
ISS, median (IQR)	17.5(9.0–28.3)	5.0(4.0–10.0)	<0.001
<16	13(36.1)	849(83.1)	<0.001
16–24	11(30.6)	126(12.3)	0.004
≥25	12(33.3)	47(4.6)	<0.001
LOS (days)	16.4 ± 15.1	7.8 ± 9.0	0.002
ICU, n (%)	20(55.6)	214(20.9)	<0.001
Mortality, n (%)	2(5.6)	6(0.6)	0.028

AIS = Abbreviated Injury Scale; ICU = intensive care unit; IQR = interquartile range; ISS = injury severity score; LOS = length of stay.

**Table 2:** Associated injuries in the body regions of patients with SIH.

Variables	Associated injuries
Head trauma, n (%)	
Epidural hematoma (EDH)	8 (22.2)
Subdural hematoma (SDH)	10 (27.8)
Subarachnoid hemorrhage (SAH)	7 (19.4)
Intracerebral hematoma (ICH)	1 (2.8)
Cerebral contusion	6 (16.7)
Maxillofacial trauma, n (%)	
Maxillary fracture	4 (11.1)
Mandibular fracture	1 (2.8)
Thoracic trauma, n (%)	
Hemothorax	1 (2.8)
Pneumothorax	3 (8.3)
Hemopneumothorax	1 (2.8)
Lung contusion	2 (5.6)
Abdominal trauma, n (%)	
Hepatic injury	5 (13.9)
Splenic injury	4 (11.1)
Renal injury	2 (5.6)
Lumbar vertebral fracture	1 (2.8)
Extremity trauma, n (%)	
Humeral fracture	1 (2.8)
Radial fracture	7 (19.4)
Ulnar fracture	3 (8.3)
Pelvic fracture	5 (13.9)
Femoral fracture	7 (19.4)
Tibia fracture	5 (13.9)
Fibular fracture	5 (13.9)

### Patient outcomes

Patients with SIH had a significantly longer LOS in the hospital (16.4 vs. 7.8 days, p = 0.002) and higher rates of ICU admission (55.6% vs. 20.9%, respectively, p < 0.001) than those with NDN (Table 1). The mortality rate was significantly higher in patients with SIH than those with NDN (5.6% vs. 0.6%, p = 0.028). However, in the developed 24 pair well-balanced propensity score-matched patient populations, who did not present with significant differences in sex, age, and ISS (Table 3), no significant difference was observed in patient outcomes in terms of LOS in the hospital (13.5 vs. 9.0 days, p = 0.099), rate of ICU admission (50.0% vs. 37.5%, p = 0.561), and in-hospital mortality rate (4.2% vs. 8.3%, p = 0.982) in pediatric patients with trauma who presented with SIH and NDN (Table 4). For the commonly-seen complications, the patients with SIH had a higher rate of pneumonia than those patients with NDN (5.6% vs. 0.4%, p < 0.001). However, these pneumonia occurred as ventilator-related complication and was only found in two patients with SIH and 4 patients with NDN (Table 5).

**Table 3:** Controlled variables (sex, age, and injury severity score [ISS]) in the developed well-balanced propensity-score matched cohort.

SIH vs. NDN	Propensity-score matched cohort			
	SIH (n = 24)	NDN (n = 24)	OR (95% CI)	P
Sex, n (%)				0.517
Male	16(66.7)	19(79.2)	0.5(0.14–1.93)	
Female	8(33.3)	5(20.8)	1.9(0.52–6.97)	
Age, years	13.5 ± 5.2	13.5 ± 5.2	—	1.000
ISS, median (IQR)	13.0(5.0–24.3)	13.0(5.0–24.3)	—	1.000

IQR = interquartile range; ISS = injury severity score; NDN = nondiabetic normoglycemia; OR = odds ratio; SIH = stress-induced hyperglycemia.

**Table 4:** Adjusted outcomes of pediatric patients with trauma who presented with SIH compared with those with NDN in the created propensity-score matched cohort.

SIH vs. NDN	Propensity-score matched cohort			
	SIH (n = 24)	NDN (n = 24)	OR (95% CI)	P
LOS (days)	15.5 ± 16.3	9.0 ± 9.6	—	0.099
ICU, n (%)	12(50.0)	9(37.5)	1.7(0.53–5.27)	0.561
Mortality, n (%)	1(4.2)	2(8.3)	0.5(0.04–5.66)	0.982

IQR = interquartile range; ISS = injury severity score; NDN = nondiabetic normoglycemia; OR = odds ratio; SIH = stress-induced hyperglycemia.

**Table 5:** Common intensive care unit related complications in these pediatric trauma patients with stress-induced hyperglycemia (SIH) and non-diabetic normoglycemia (NDN).

Variables	SIH (n = 36)	NDN (n = 1,022)	P
Pneumonia	2 (5.6)	4 (0.4)	<0.001
Urinary tract infection	1 (2.8)	9 (0.9)	0.248
Wound infection	0 (0.0)	5 (0.5)	0.674
Acute respiratory failure	0 (0.0)	3 (0.3)	0.745
Upper gastrointestinal bleeding	0 (0.0)	2 (0.2)	0.790
Fat embolism	0 (0.0)	2 (0.2)	0.790

## Discussion

This study revealed that the pediatric trauma patients who presented with SIH had a significantly poor outcome than those with NDN. However, after controlling for sex, age, and ISS, no significant difference was observed in the outcomes in terms of length of hospital stay, rate of ICU admission, and in-hospital mortality rate between pediatric patients with trauma who presented with SIH and NDN, indicating that the different baseline characteristics of the patients, particularly injury severity, may be associated with poor outcomes (a longer hospital stay as well as a higher rate of ICU admission and in-hospital mortality) in pediatric patients with trauma who presented with SIH but not in those with NDN. In adult patients with trauma, a positive correlation between SIH and poor outcome was observed even after adjusting the baseline difference of injury severity^15–17^. In pediatric patients with trauma, it had been reported that a 50% mortality rate was found if there is an extreme stress hyperglycemia (>300 mg/dL)^14^ and the presence of SIH was a marker of poor outcome and was associated with an extremely high mortality rate^22^. However, according to the results of this study, the impact of SIH on mortality is different between the pediatric and adult patients with trauma. After controlling baseline difference of sex, age, and injury severity, SIH was not associated with a higher mortality in the pediatric patients with trauma. In addition, although in severely injured children, hyperglycemia had been reported to be correlated to an increased incidence of infection^23^ and organ dysfunction^24^, only the rate of pneumonia was significantly higher in pediatric trauma patients with SIH than those patients with NDN. Furthermore, in this study, 36 of 1,058 (2.84%) non-diabetic pediatric patients with trauma had SIH. In comparison with our prior published article that 493 of 8,299 (5.94%) non-diabetic adult trauma patients had SIH^15^, albeit the difference of injury severity was not considered. The incidence of SIH is lower in the pediatric patients than the adult patients. This study also indicated that, upon major trauma, the response of pediatric patients with SIH is different from that of adult patients.

In this study, head trauma was most frequently associated with SIH. It had been reported that hyperglycemia in children with traumatic brain injury is associated with increased mortality, prolonged duration of mechanical ventilation, and longer ICU stay^25^. An elevated serum glucose level >200 mg/dL^26^ or hyperglycemia that lasted beyond the initial 48 hours^27^ is associated with a higher mortality in children with traumatic brain injury. In this study, significantly higher rates were observed for the patients with an AIS score ≥3 in head/neck, thorax, abdomen, and external injuries in patients with SIH compared with those with NDN. However, after excluding those patients with polytrauma (AIS ≥3 in at least two body regions), in those patients with isolated AIS ≥3, a higher rate was only observed in external injury, but no in head/neck, thorax, or abdomen injuries, in the patients with SIH than those with NDN. This result indicated that a higher ISS of these pediatric trauma patients with SIH than those with NDN is usually associated with polytrauma. Therefore, the poor outcomes (i.e. a longer hospital stay as well as a higher rate of ICU admission and in-hospital mortality) of the pediatric trauma patients with SIH than the patients with NDN would rather be attributed to the associated higher ISS but not the noted higher rate of head trauma.

The present study had some limitations. First, a retrospective design might have caused selection bias. Second, the patients who were declared dead at the scene of the accident or upon arrival at the ED were not included in the trauma data; this may be a source of selection bias on the assessment of mortality outcome. Third, stress might also induce hyperglycemia in patients, and confounding factors, such as the type of injury (penetrating or burn), traumatic brain injury, and physiologic parameter (change of base excess) upon admission, were not analyzed due to a relatively small sample size in this study. The small sample size might have also impeded a powerful statistical analysis. Fourth, the appropriate glucose targets and strategies for sugar control have been inconclusive and may vary among different patients. Therefore, we could only assume that all patients had received uniform management in the clinical setting. Finally, results derived from one single trauma center may not be generalized to other regions or countries.

## Conclusions

No significant difference was observed in the outcomes in terms of LOS in the hospital, rate of ICU admission, and in-hospital mortality rate among pediatric patients with trauma who presented with SIH and NDN, after controlling for sex, age, and ISS. The different baseline characteristics of the patients, particularly injury severity, may be associated with poor outcome in pediatric trauma patients with SIH compared with those with NDN. The study revealed that the poor outcomes which were associated with SIH in adult trauma patients was not found in the pediatric trauma patients.

### Compliance with ethical standards

All procedures performed in studies involving human participants were in accordance with the ethical standards of the institutional and/or national research committee and with the 1964 Helsinki declaration and its later amendments or comparable ethical standards. Informed consent was waived according to IRB regulations.
